# Supplementary material for: Marine Organisms from the Yucatan Peninsula (Mexico) as a Potential Natural Source of Antibacterial Compounds
Source: Mar Drugs. 2020 Jul 18;18(7):369. doi: 10.3390/md18070369 (PMC7404059; doi:10.3390/md18070369)
Supplement: Supplementary file 1 [file marinedrugs-18-00369-s001.pdf]

Supplementary Materials

Marine organisms from the Yucatan Peninsula (Mexico) as a potential natural source of antibacterial compounds

Dawrin Pech-Puch [1], Mar Pérez-Povedano [1], Patricia Gómez [2], Marta Martínez-Guitián [3], Cristina Lasarte-Monterrubio [3], Juan Carlos Vázquez-Ucha [3], María Lourdes Novoa-Olmedo [3], Sergio Guillén-Hernández[4], Harold Villegas-Hernández[4], Germán Bou [3], Jaime Rodríguez [1,*], Alejandro Beceiro [3] and Carlos Jiménez [1,*]

[1] Centro de Investigacións Científicas Avanzadas (CICA) e Departmento de Química, Facultade de Ciencias, AE CICA-INIBIC, Universidade da Coruña, 15071 A Coruña, Spain. dawrin.j.pech@udc.es (D.P.-P.); perezpovedanomaranabel@gmail.com (M.P.-P.)

[2] Unidad Académica de Ecología y Biodiversidad Acuática, Instituto de Ciencias del Mar y Limnología, Universidad Nacional Autónoma de México, 04510, CDMX, México. patricia@cmarl.unam.mx (P.G.)

[3] Servicio de Microbioloxía. Instituto de Investigación Biomédica AE CICA-INIBIC, Complexo Hospitalario Universitario A Coruña, 15006, A Coruña, Spain. m.martinez.guitian@gmail.com (M.M.-G.); crlasarm@gmail.com (C.L.-M.); juan.vazquez@udc.es (J.C.V.-U.) marianovoa@usb.ve (M.L.N.-O.); German.Bou.Arevalo@sergas.es (G.B.); Alejandro.Beceiro.Casas@sergas.es (A.B.)

[4] Departamento de Biología Marina. Campus de Ciencias Biológicas y Agropecuarias. Universidad Autónoma de Yucatán. Carretera Mérida-Xmatkuil Km. 15.5, C.P. 97000, Mérida, Yucatán, México. ghernand@correo.uady.mx (M.G.-H.); harold.villegas@correo.uady.mx (H.V.-H.)

* Correspondence: jaime.rodriguez@udc.es (J.R.); carlos.jimenez@udc.es, (C.J.)

INDEX

Figure S1. Chromatogram of fraction R4 of *Amphimedon compressa*…...………………..………S3

Figure S2-S3. NMR spectra data of subfraction R4H2 in CD_3OD…….……....…………......S4

Figure S4. Chromatogram of fraction R2 of *Agelas citrina*….…………..……………...….....S5

Figure S5 (+)-HRESIMS of (-)-agelasine B ..…………………………...……………….…….S6

Figure S6-S7. NMR spectra data of (-)-agelasine B in DMSO-d6……………..…………......S7

Table S1. Description of bacterial type strains used………………..………...…...….........S8

Table S2. MICs of antibiotics against bacterial pathogens………………..………………..S9

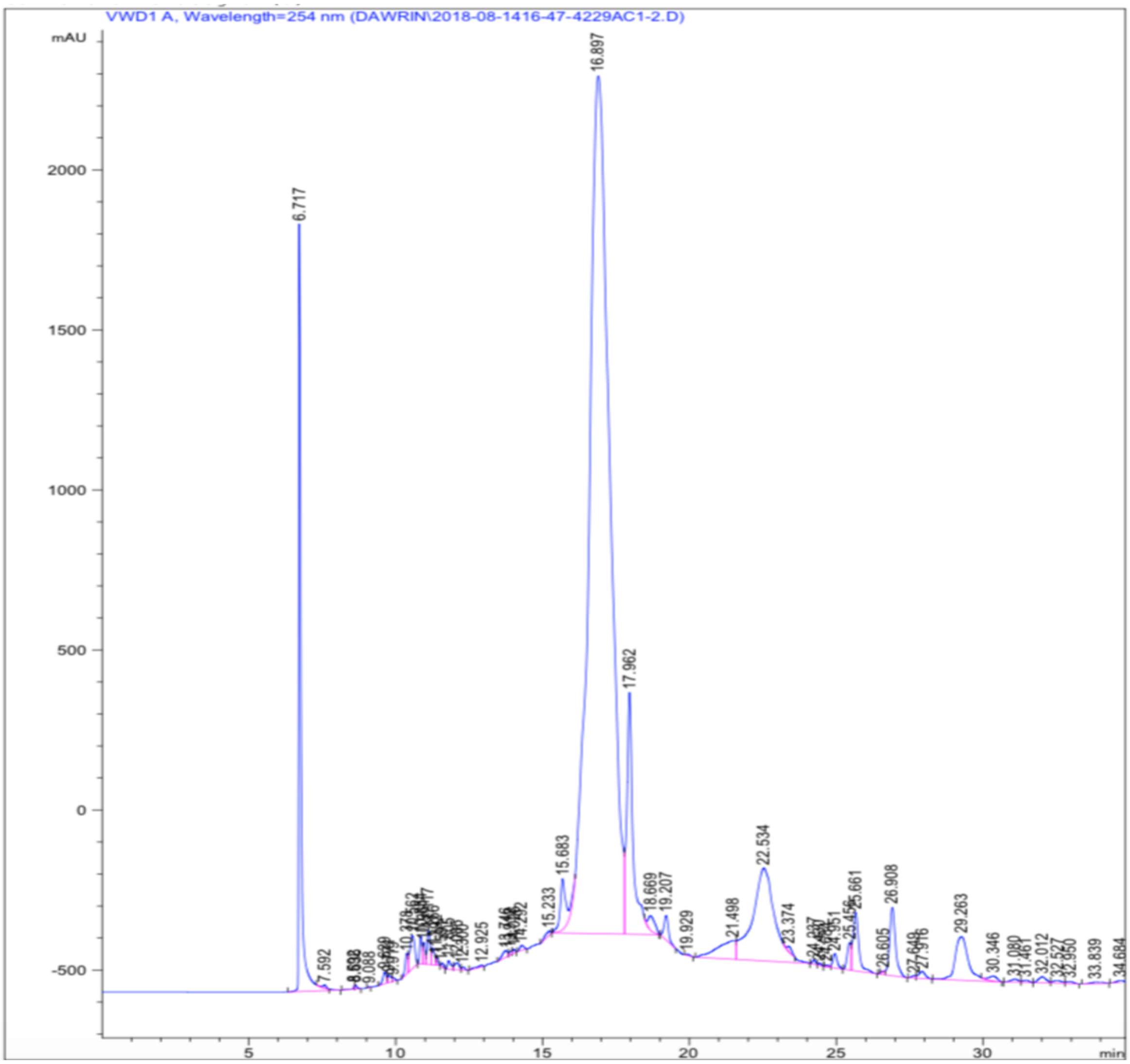

Figure S1. RP-HPLC chromatogram of the SPE fraction R4 from the sponge *Amphimedon compressa*.

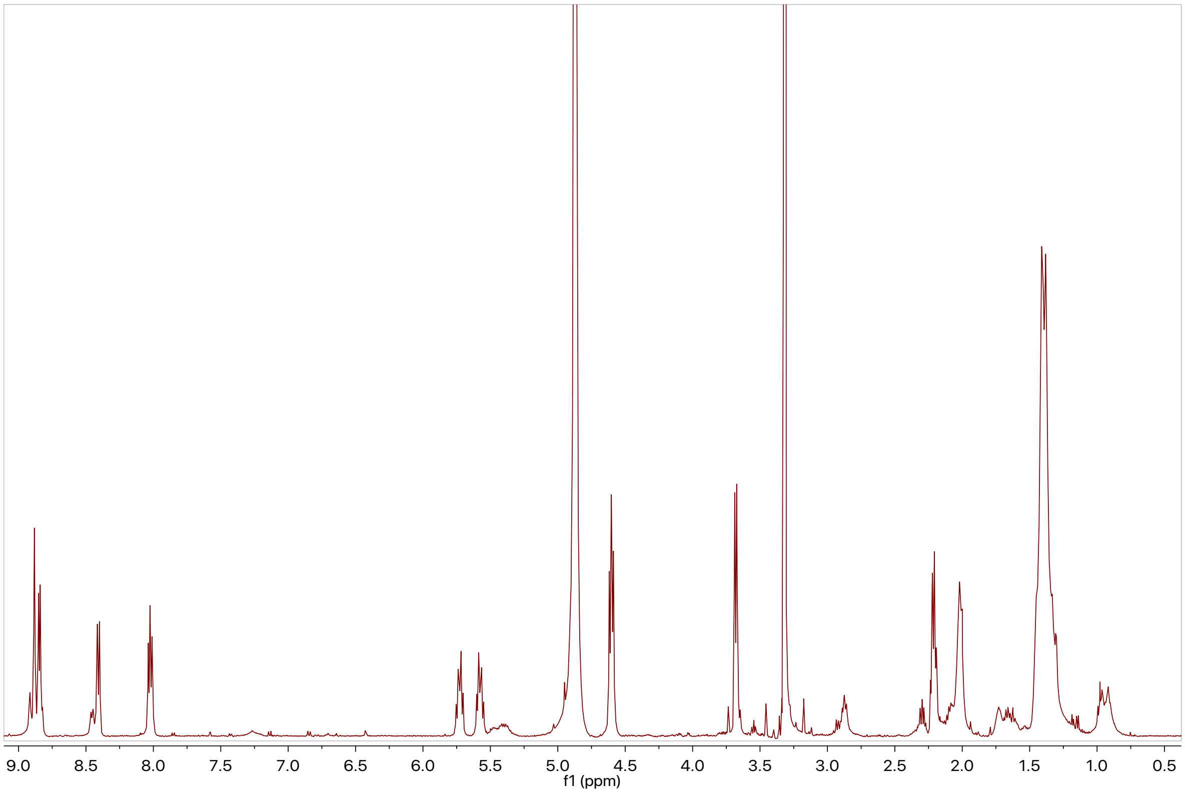

Figure S2. ^{1}H NMR spectrum of the R4H2 subfraction (500 MHz, CD_3OD).

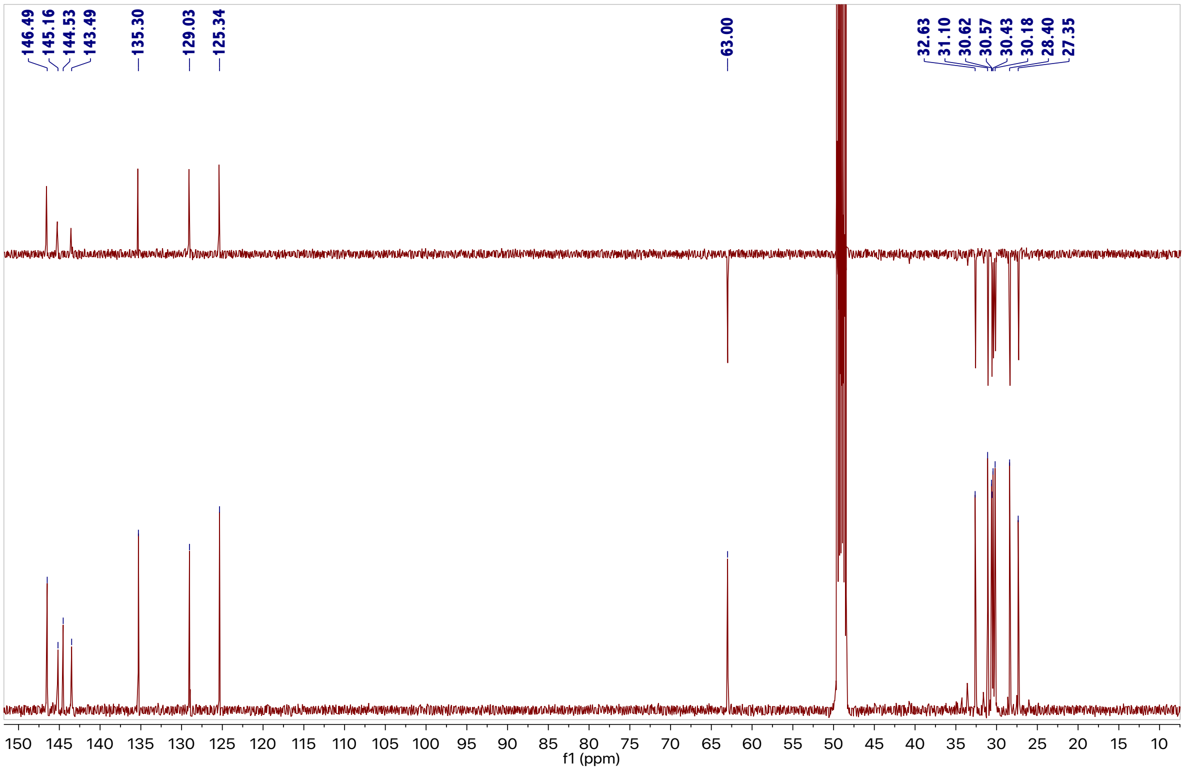

Figure S3. ^{13}C NMR and DEPT-135 spectra of the R4H2 subfraction (125 MHz, CD_3OD).

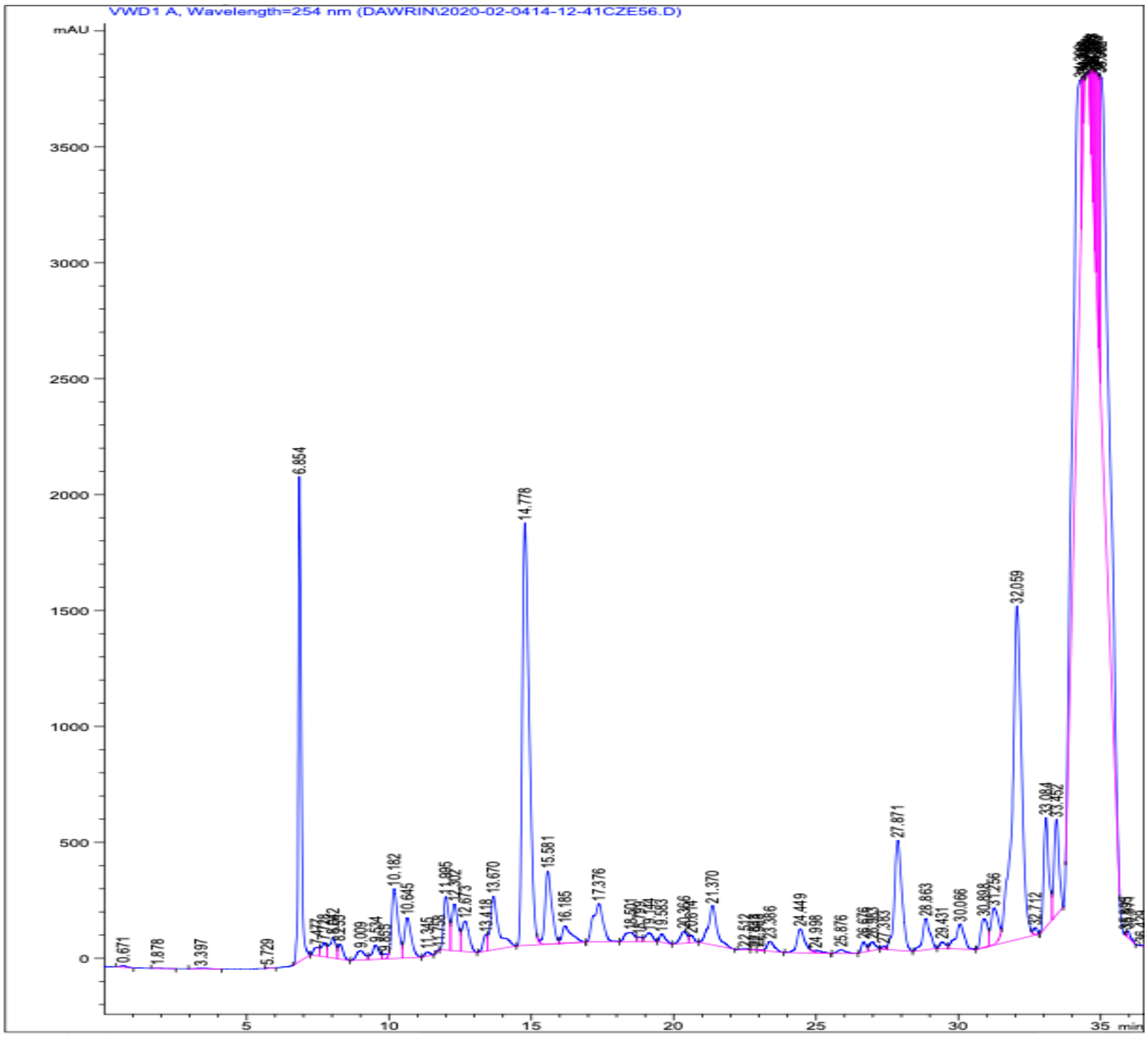

Figure S4. RP-HPLC chromatogram of the SPE subfraction R2 from the sponge *Agelas citrina.*

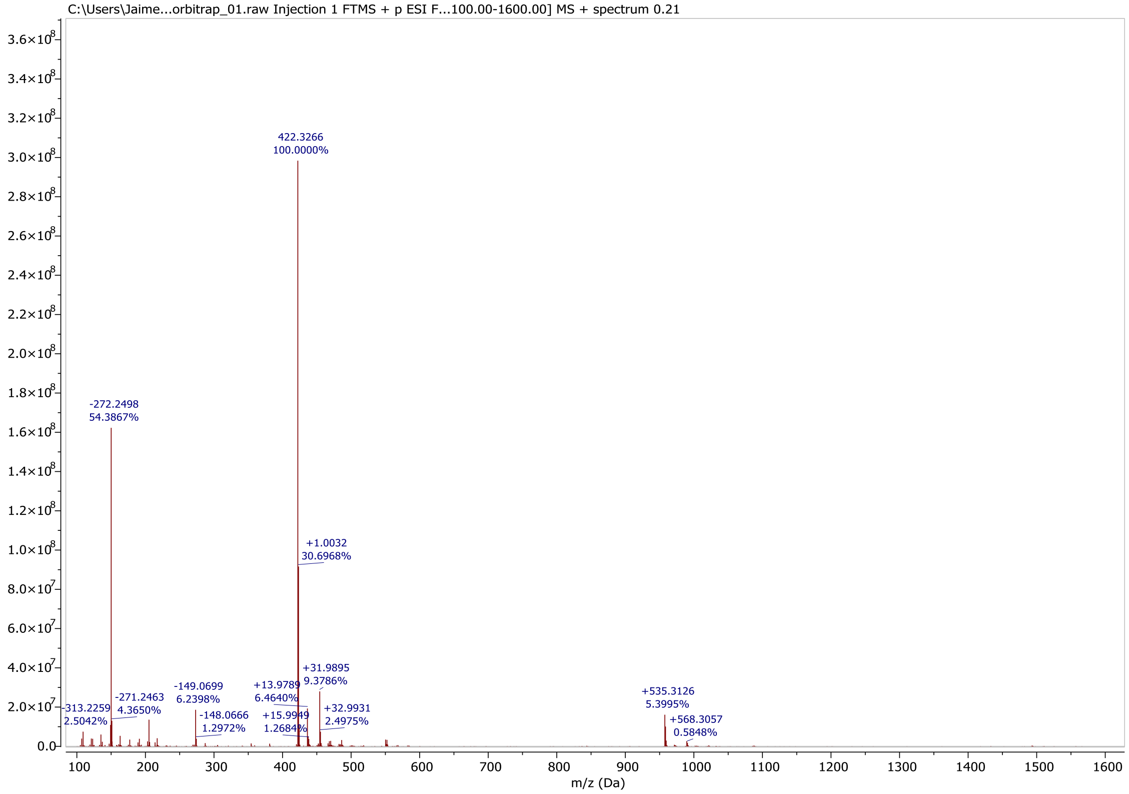

Figure S5. (+)-HRESIMS of agelasine B.

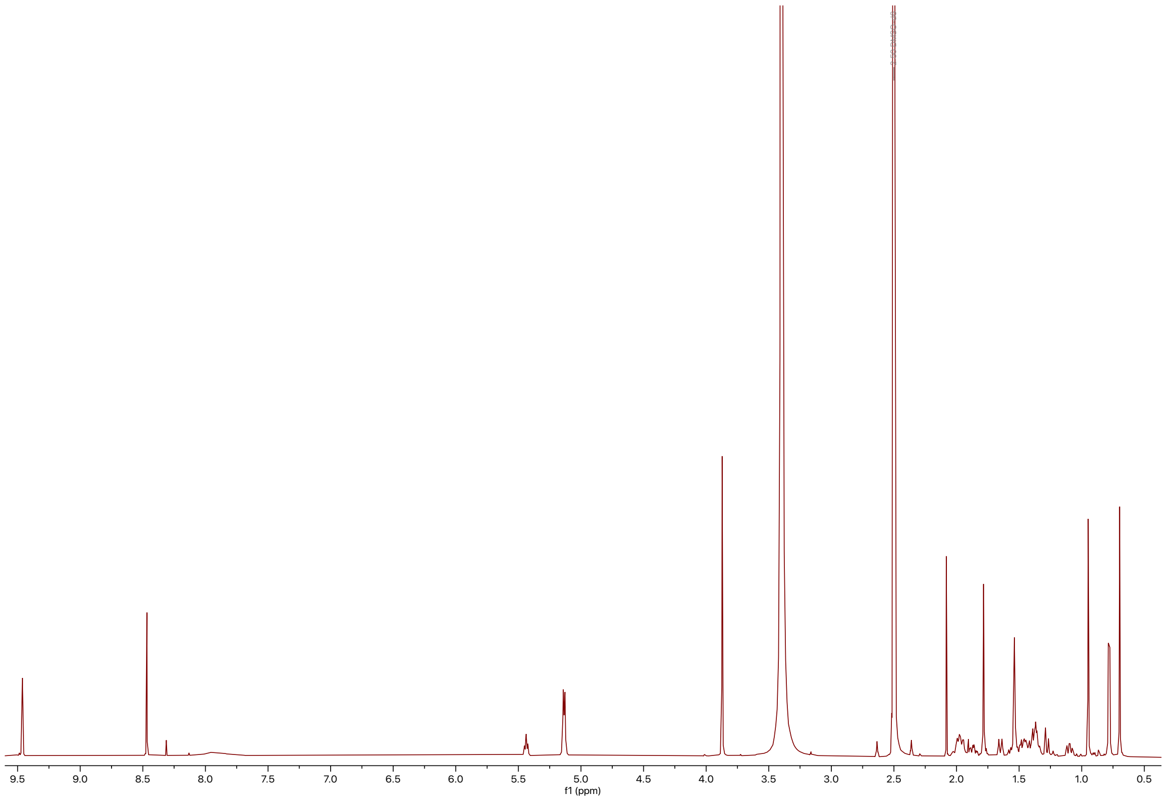

Figure S6. ^{1}H NMR spectrum of agelasine B (500 MHz, DMSO-d6).

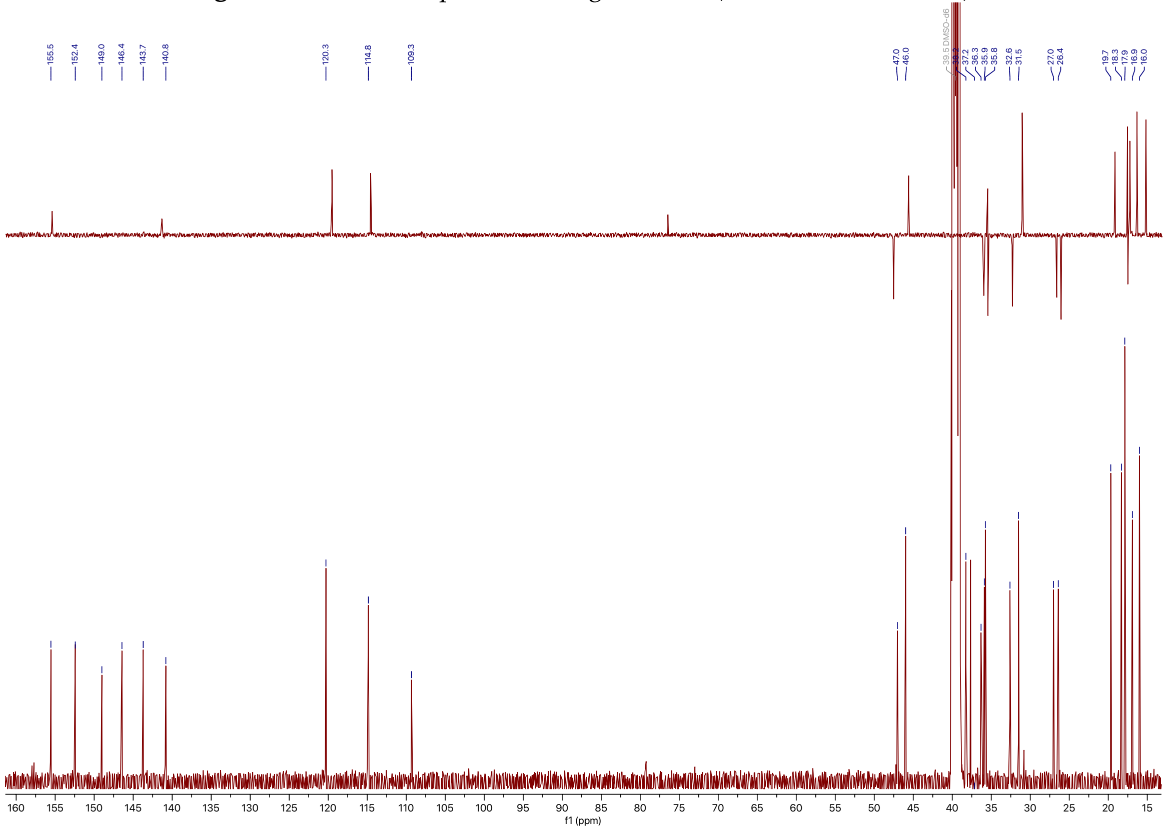

Figure S7. ^{13}C NMR and DEPT-135 spectra of agelasine B (125 MHz, DMSO-d6).

Table S1. Description of bacterial type strains used.

**Bacterial sp.**	**Strains**	**Description**
A. baumannii	ATCC [a] 17978<br><br>ABRIM (clinical isolate)	Gram-negative rod-shaped bacterium, can produce pneumonia, blood infections, wound and surgical site infections and urinary tract infections.[1]
K. pneumoniae	ATCC 700603<br><br>Kp3380 (clinical isolate)	Gram-negative rod-shaped encapsulate bacterium, produces pneumonia, urinary tract infections, sepsis, meningitis, diarrhea, and soft tissue infections [2]
P. aeruginosa	ATCC 27853<br><br>PAO1 (reference strain)	Gram-negative, encapsulated, rod-shaped bacterium. Infects the airway, urinary tract, burns and wounds, and also causes other blood infections.[3]
S. aureus	ATCC 29213<br><br>USA LAC 300 (clinical isolate)	Gram-positive, round-shaped bacterium, one of the most common causes of bacteraemia and infective endocarditis and can also cause various skin and soft-tissue infections.[4]

[a] American Type Culture Collection

[1] Antunes, L.C.S.; Visca, P.; Towner, K.J. *Acinetobacter baumannii*: evolution of a global pathogen. *Pathog. Dis*. **2014**, *71*, 292-301

[2] Podschun, R.; Ullmann, U. *Klebsiella* spp. as Nosocomial Pathogens: Epidemiology, Taxonomy, Typing Methods, and Pathogenicity Factors. *Clin. Microbiol. Rev.* **1998**, 11, 589–603.

[3] Bassetti, M.; Vena, A.; Croxatto, A.; Righi, E.; Guery, B. How to manage *Pseudomonas aeruginosa* infections. *Drugs in Context* **2018**, *7*, 1–18.

[4] Tong, S.Y.C.; Davis, J.S.; Eichenberger, E.; Holland, T.L.; Fowler, V.G. *Staphylococcus aureus* Infections: Epidemiology, Pathophysiology, Clinical Manifestations, and Management. *Clin. Microbiol. Rev.* **2015**, *28*, 603–661.
